# Informed consent in oncology clinical trials: A Brown University Oncology Research Group prospective cross-sectional pilot study

**DOI:** 10.1371/journal.pone.0172957

**Published:** 2017-02-24

**Authors:** Andrew Schumacher, William M. Sikov, Matthew I. Quesenberry, Howard Safran, Humera Khurshid, Kristen M. Mitchell, Adam J. Olszewski

**Affiliations:** The Brown University Oncology Research Group, Providence, Rhode Island, United States of America; University of Liverpool, UNITED KINGDOM

## Abstract

**Background:**

Informed consent forms (ICFs) for oncology clinical trials have grown increasingly longer and more complex. We evaluated objective understanding of critical components of informed consent among patients enrolling in contemporary trials of conventional or novel biologic/targeted therapies.

**Methods:**

We evaluated ICFs for cancer clinical trials for length and readability, and patients registered on those studies were asked to complete a validated 14-question survey assessing their understanding of key characteristics of the trial. Mean scores were compared in groups defined by trial and patient characteristics.

**Results:**

Fifty patients, of whom half participated in trials of immunotherapy or biologic/targeted agents and half in trials of conventional therapy, completed the survey. On average, ICFs for industry-originated trials (N = 9 trials) were significantly longer (P < .0001) and had lower Flesch ease-of-reading scores (P = .003) than investigator-initiated trials (N = 11). At least 80% of patients incorrectly responded to three key questions which addressed the experimental nature of their trial therapy, its purported efficacy and potential risks relative to alternative treatments. The mean objective understanding score was 76.9±8.8, but it was statistically significantly lower for patients who had not completed high school (P = .011). The scores did not differ significantly by type of cancer therapy (P = .12) or trial sponsor (P = .38).

**Conclusions:**

Many participants enrolled on cancer trials had poor understanding of essential elements of their trial. In order to ensure true informed consent, innovative approaches, such as expanded in-person counseling adapted to the patient’s education level or cultural characteristics should be evaluated across socio-demographic groups.

**Trial registration:**

Clinicaltrials.gov NCT01772511

## Introduction

Ensuring patient autonomy and minimizing harm by obtaining informed consent is an essential prerequisite to conducting clinical trials, as delineated in the Declaration of Helsinki and other foundational documents for research ethics [1, 2]. In oncology, the consenting process is complicated by patient anxiety in the face of a life-threatening o disease, and by the fact that participation in a trial may be the only way to gain access to novel, potentially more promising therapies. Comprehension of research design and risks may also be compromised when a patient is acutely ill or suffering from a chronic, debilitating disease [3]. However, to meet the obligations of true informed consent, a thorough appreciation of the potential benefits, toxicities and alternatives to experimental therapy is of paramount importance. Increasing complexity of trial designs, the availability of novel agents with remarkable efficacy but only in highly selective patient subgroups, and extensive media coverage of “personalized medicine” or “genomic breakthroughs” may exacerbate a patient’s therapeutic misconception by creating unrealistic expectations of benefit.

A clinical trial’s informed consent form (ICF) is supposed to provide complete and clear information about the potential risks and benefits of participation in that trial, but the ICFs for cancer clinical trials have become increasingly complex and lengthy [4, 5]. To a large extent, their content is shaped by regulatory and legal language designed to protect the research institution and/or sponsor rather than trial subjects [4]. As a result, patients may not achieve the level of understanding that is vital to the provision of informed consent, as has been noted in multiple studies over the past 20 years [6–9]. This can occur despite patient reports of satisfaction with the informed consent process and perception of knowledge. In some studies of the consenting process, subjects declared receipt of sufficient and comprehensible information, even though their objective understanding of the details of the study to which they had agreed were lacking [9–11].

Prior studies have investigated the effects of various approaches to enhancing overall understanding of the ICF, primarily by innovative modes of delivery, but improvements have not been consistently demonstrated.[12–14] Some studies reported that certain knowledge domains, like details of treatment and its duration, or randomization procedures, were particularly poorly understood, but did not specifically investigate which domains are at higher risk.[7, 15] Despite their length, ICF’s often omit significant information important for the informed consent process.[5] Identification of what information contained in the ICF is most likely to be poorly understood may help to focus further work on updating ICFs to improve their functionality.

We hypothesized that despite years of effort, and with the increased complexity of oncologic therapy, currently used ICFs may not promote adequate understanding of critical study-related information for many patients, and that levels of such understanding may vary by patient or trial characteristics. The primary purpose of this pilot study was thus to evaluate whether participants in oncology clinical trials objectively comprehend the basic elements of the studies, and to identify which domains are most at risk of being misunderstood. The secondary objective was to evaluate how patient- (age, sex, race, education level) or trial- (sponsorship by an academic investigator, cooperative group, or pharmaceutical industry, type of cancer therapy) related factors influence the level of understanding.

## Patients and methods

We conducted a prospective observational cross-sectional study. Patients were enrolled between June 2012 and October 2014. The study was approved by the Rhode Island Hospital and The Miriam Hospital Institutional Review Boards (IRB) and registered at clinicaltrials.gov as NCT01772511 (see S1 Appendix, S1 Dataset). All subjects provided a written informed consent for participation. Patients were eligible if they were 18 years of age or older, English-speaking (though not necessarily as the first language), and if they had agreed to participate in a trial of cancer therapy at the Rhode Island Hospital Comprehensive Cancer Centers (RIHCCC). Rhode Island Hospital is an academic medical center affiliated with the Warren Alpert Medical School of Brown University, which offers investigator-initiated, cooperative group, and pharmaceutical industry-sponsored phase 1, 2 and 3 trials, enrolling about 200 patients annually. Investigator-initiated trials are authored by one or more faculty members of the Brown University Division of Hematology-Oncology and coordinated by the Brown University Oncology Group (BrUOG). Qualifying trials could involve treatment with experimental chemotherapy, radiation therapy, targeted, biologic or endocrine agents. Eligible subjects were identified by the clinical research staff and offered participation after they had completed the informed consent process for their primary treatment trial and signed the relevant ICF. This pilot study enrolled a convenience sample without a pre-determined cohort size.

### Outcome measures and procedures

ICF readability was assessed using the Flesch-Kincaid reading grade level and Flesch Reading Ease Score [16, 17]. These commonly employed scales use word and sentence length to calculate the education level (US grade level) necessary to understand a piece of text and its ease of reading. The reading ease is scored between 0 and 100, with increasing score indicating easier read. A score of 60–70 is considered to be “plain English”, 50–60 “fairly difficult”, and 30–50 “difficult to read”.

In order to measure patients’ objective understanding of the informed consent components, we used a modified version of the Quality of Informed Consent (QuIC) survey. The QuIC has been designed to evaluate the 8 basic elements of the ICF as identified by the United States (US) Code of Federal Regulations (45 CFR 46.116(a) and 21 CFR 50.25): 1) explanation of the purpose and procedures of the research, 2) description of foreseeable risks or discomforts, 3) benefits to self and others, 4) disclosure of alternative treatments, 5) confidentiality of records, 6) explanation of compensation or treatment in the event of research-related injury, 7) contact information in case of research-related questions or injury, and 8) a statement reinforcing voluntary participation [18]. The QuIC contains 34 questions measuring subject’s objective (Part A, 20 items) and subjective (Part B, 14 items) understanding of the research, and has been validated in a survey of oncology clinical trials, with an average completion time of 7.2 minutes [11, 18]. We used a condensed version of the QuIC Part A (objective understanding), eliminating 6 questions specific to clinical trial phase. The 14 remaining items were applicable regardless of trial design and addressed the basic elements of the informed consent. Each question was answerable with a triple-bounded binary choice format: “Agree”, “Disagree” or “Unsure”, and scored as correct (100 points), incorrect (0 points) or unsure (50 points). Nine questions were phrased positively (i.e. “Agree” being the correct answer), and 5 were phrased negatively to avoid agreement bias. The primary endpoint of the study was the summary QuIC-A score. All participants filled out the study survey in person, during a clinical visit, without a time limitation. There was no further follow up for subjects on this study, and no specific incentives offered to participants.

### Statistical analysis

Descriptive statistics are reported with means and standard deviations for continuous variables, or percentages for categorical variables. The statistics for different consent versions in each clinical trial were averaged and analyzed on a per-trial basis, and compared using one-way ANOVA. The QuIC-A score was expressed on a normalized scale (0 to 100), thus representing a subject’s average objective understanding of the informed consent components. Although no validated threshold of what constitutes an “adequate” score has been established in the literature, and any departure from full understanding (i.e. score 100) might indicate a deficient informed consent, we compared our results from prior studies using the QuIC-A score, and between groups of interest. For the secondary objective, an exploratory analysis of factors judged by the investigators to be potentially associated with the QuIC-A score was envisaged, including age, native language other than English, level of education, cancer type, phase of clinical trial, type of sponsor, and specific hospital as potential explanatory variables. We assessed association between the QuIC-A scores (continuous variable) and each explanatory variable using Pearson’s correlation coefficients (for other continuous variables) or one-way ANOVA (for categorical variables). Normality of distribution was confirmed in all cases by Shapiro-Wilk test. Potential moderation of the effect of readability by education level was evaluated by a test of interaction between those variables in a linear regression model.[19] In this exploratory analysis with a limited sample size and explanatory variables defined a priori, we did not perform adjustment for multiple testing.[20] All statistical tests were two-sided, with α of 0.05 considered significant, and were conducted using Stata/MP 14.1 (StataCorp LP, College Station, TX).

## Results

**Fig 1:**
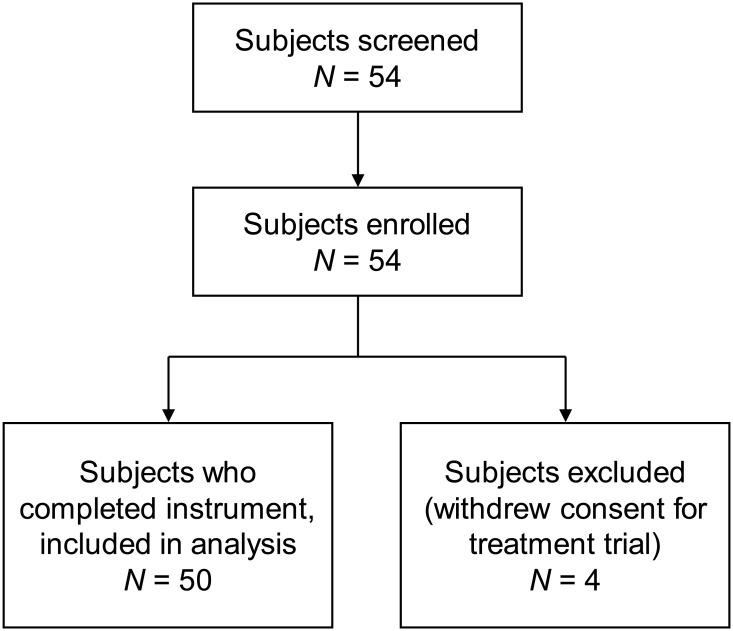
CONSORT diagram for flow of study participants.

The study enrolled 54 participants treated on 26 different clinical trials (Fig 1, Table 1). Four patients did not complete the survey because they withdrew from their treatment trial beforehand and were excluded from the analysis, whereas 50 subjects (93%) completed the QuIC-A questionnaire. Median age was 61 years (range, 35 to 85). Most patients were white, native English speakers, and half had some college education. Non-white patients self-reported as African American or Portuguese Hispanic. A majority of patients in this cohort had advanced gastrointestinal tumors. Fifty percent enrolled in clinical trials studying biologic therapy (monoclonal antibodies or antibody-drug conjugates), immunotherapy (vaccines or immune checkpoint inhibitors) or oral targeted agents (tyrosine kinase inhibitors).

**Table 1:** Characteristics of the study participants.

Variable	Level	*N*	(%)
All patients		54	
Age	<60 years	25	(46%)
	≥60 years	29	(54%)
Sex	Women	24	(44%)
	Men	30	(56%)
Race	White	51	(94%)
	Non-white	3	(6%)
Education level	Less than high school diploma	5	(9%)
	High school diploma	22	(41%)
	Associate degree	7	(13%)
	Bachelor’s degree	14	(26%)
	Master’s degree or higher	6	(11%)
Native language	English	52	(96%)
	Other	2	(4%)
Cancer site	Pancreas	20	(37%)
	Colorectal	12	(22%)
	Other gastrointestinal	4	(8%)
	Central nervous system	4	(7%)
	Breast	4	(7%)
	Lung	4	(7%)
	Prostate	2	(4%)
	Renal	2	(4%)
	Head/neck	1	(2%)
	Melanoma	1	(2%)
Type of clinical trial	Investigator-initiated	28	(52%)
	Industry-sponsored	17	(31%)
	Cooperative group	9	(17%)
Phase of clinical trial	1	21	(39%)
	2	19	(35%)
	3	14	(26%)
Trial therapy	Conventional therapy ^a^	27	(50%)
	Monoclonal antibody or ADC	13	(24%)
	Immunotherapy	9	(17%)
	Targeted therapy	5	(9%)

^a^ including cytotoxic chemotherapy, endocrine therapy or radiation therapy

ADC: antibody-drug conjugate

Among the 26 trials, 9 (35%) were phase 1, 9 (35%) were phase 2, and 8 (31%) were phase 3 clinical trials. Eleven studies (42%) were investigator-initiated, 6 (23%) were cooperative group-sponsored, and 9 (35%) were industry-sponsored. Nine studies (35%) involved only cytotoxic chemotherapy and/or radiation therapy, while 17 (65%) included administration of biologic, targeted or immune-directed agents.

Mean time between consent for the treatment trial and for the current study was 60 ±51 days. Mean length of the ICF was 16.6 ±5.7 pages, and was significantly longer for phase 3 trials (21.4 ±3.8 pages) than for phase 1 (15.5 ±4.8) or phase 2 (13.5 ±5.5) trials (*P* = .0069). The average length of the ICF was also nearly twice as long for industry-sponsored (*N* = 9, 20.2 ±2.8 pages) or cooperative group-sponsored (*N* = 6, 21.5 ±3.5) trials than for investigator-initiated trials (*N* = 11, 11.0 ±2.8 pages, *P* < .0001). Mean reading grade required to understand the ICF was 11.2 ±0.7, and mean Reading Ease Score was 50.2 ±3.1, placing it at the border of the “fairly difficult” and “difficult” categories. The ICFs for industry-sponsored trials had a slightly higher required reading grade (11.7 ±0.7 compared with 11.0 ±0.7 for investigator-initiated and 10.8 ±0.6 for cooperative group trials, *P* = .025), and a significantly worse Reading Ease Score (mean, 47.9 ±3.0, compared with 50.8 ±2.3 for investigator-initiated and 52.9 ±2.0 for cooperative group trials, *P* = .003). Consents for trials of biologic, targeted, or immunotherapy agents were significantly longer (18.5 ±4.9 pages) than for trials of conventional therapies (13.0 ±5.5 pages, *P* = .016), but the difference disappeared after adjusting for the type of trial sponsor (*P* = .63). There was no statistically significant difference in mean reading grade (*P* = .22) or Reading Ease Score (*P* = .15) depending on the type of antineoplastic agent.

**Fig 2:**
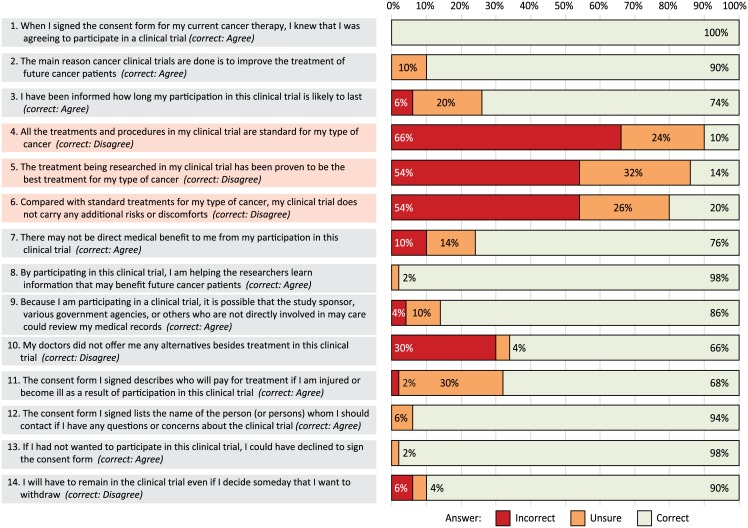
Percent of correct and incorrect responses to each survey question.

The results of the objective understanding survey (QuIC-A) are shown in Fig 2. Mean QuIC-A summary score was 76.9 ±8.8. A single missing response was scored as incorrect (with a sensitivity analysis scoring it as “correct”, showing no difference in results). Participants gave mostly correct answers to questions related to the general understanding of participation in research, purpose of the research, confidentiality of records, contacting the research team, and the voluntary nature of participation (>85% correct). In contrast, their understanding of the domains related to the experimental therapies was very poor. Over 80% of the participants did not recognize that the treatment being administered on their clinical trial was experimental and non-standard, that the benefits of treatment were uncertain, and that participation was associated with additional risks. A third of patients did not understand that alternative treatments were available, or were unsure about available support in case of research-related injury.

Mean QuIC-A score did not statistically significantly differ by age (*P* = .33), sex (*P* = .33), phase of the clinical trial (*P* = .54) or its sponsor type (*P* = .38), and did not correlate with the ICF length (*P* = .11), reading grade (*P* = .63) or Reading Ease Score (*P* = .45). It also did not correlate with time between consent for the clinical trial and for the current study (*P* = .57). There was also no significant difference between participants in trials of conventional or biologic/targeted agents (*P* = .12). However, the score was significantly lower for patients with education level less than a high school diploma (mean, 64.3 ±10.4, compared with 77.8±8.5 for high school diploma, 80.6±5.0 for associate degree, 77.2 ±6.4 for bachelor’s degree, and 79.2 ±10.0 for master’s degree; ANOVA F = 3.7, *P* = .011). We found no statistically significant interaction between the education level (high school diploma or higher) and the Flesch Reading Ease Score (*P* = .22), but the study was not sufficiently powered for a more thorough analysis of a potential effect moderation.

## Discussion

In this prospective study, we assessed comprehension of critical elements of informed consent among patients enrolled on contemporary cancer clinical trials, half of whom were receiving immunotherapy or a biologic/targeted agent. Our two main findings are that many patients exhibited a poor grasp of key consent elements related to their experimental therapy, and that overall comprehension may correlate with education level, but not with the type of trial sponsor or the type of experimental agent. These findings extend prior knowledge by demonstrating ongoing issues related to comprehension of informed consent in the era of increasing use of novel therapies, and indicating the main areas of knowledge deficiency.

Results from the QuIC-A instrument in our cohort were similar to those previously reported. The mean QuIC-A score was 77.8 (±9.4) in a cross-sectional validation study by Joffe et al., in which research participants from a tertiary center responded to a mailed survey [11]. The three QuIC-A questions related to therapeutic misconception (items 4, 5, and 6 in our version) were incorrectly answered by 75%, 69%, and 64% of subjects in that report [11]. We found an even higher rate of incorrect responses (90%, 86%, and 80%, respectively), possibly because we administered the survey in person rather than by mail, thus eliminating attrition bias from non-responders (which was 28% in Joffe et al.). Jefford et al. reported a mean QuIC-A score of 77.6 among 102 cancer patients, whereas the mean subjective score (QuIC-B) was 91.5, illustrating the discrepancy between patients’ perception of their level of understanding and their objective comprehension [9]. We further compared groups defined by type of trial sponsor, and by use of conventional or novel immunotherapeutic or targeted/biologic agents. Despite the significantly longer ICFs in cooperative group or pharmaceutical trials compared with those designed by local investigators, and despite worse readability scores of ICFs from industry-sponsored trials, we found no significant difference in the understanding score. Thus, despite the recognition of the inadequacies of ICFs used in the past, we do not appear to have made substantial progress towards doing a better job of providing our study patients with true informed consent regarding the experimental nature of their treatment and accurate estimates of its likely benefits and risks.

The actual readability of ICF’s has not improved either. In 1994, Grossman et al. analyzed 137 ICFs from oncology clinical trials conducted at The Johns Hopkins Oncology Center, reporting a mean Flesch Reading Ease Score of 52.6±8.7, and mean reading grade level of 11.1±1.7 [6]. In 2004, a review from Emory University Winship Cancer Institute found a mean Reading Ease Score of 45.5±5.2 [21]. These values are close to our updated estimates, illustrating that the ICF readability remains a major problem, especially given the reality of literacy levels in the US, which are basic for 22%, and below basic for additional 14% of Americans [22]. Food and Drug Administration (FDA) guidelines recommend presenting medical information at or below the 8^th^ grade level, and simplifying it further for subjects with low literacy [23]. Given a mean reading grade level of 11, it should come as no surprise that patients with less than high school diploma are at the highest risk of failing to absorb the information conveyed in the ICF. Another prospective study by Breese et al., conducted in a non-cancer setting, reported that low education level, race, and non-English primary language were all associated with lower comprehension scores in a univariate analysis (*P* < .0001), but only education remained significant in a multivariable model [24]. Although in our analysis the ICF length did not correlate with the QuIC score, all consent documents were long (16 pages on average). Beardsley et al. suggested that comprehension significantly improves when the length is 7 pages or less—a finding that was incorporated in the 2013 National Cancer Institute initiative to shorten the ICF templates [5].

Efforts to improve the clarity of ICFs in oncology have met numerous challenges [12]. In a comparative study, shortening the ICF and lowering the required grade level from 16 to 7 did not improve understanding of key trial design elements or associated risks, even though patients stated a preference for the shorter form [7]. In a recent randomized study by Hoffner at al., multimedia enhancements to the ICF did not lead to better comprehension, despite patient reports of satisfaction with the process [13]. Coyne et al. randomized participants in 3 trials of cancer chemotherapy to a standard or simplified ICF, and found lower anxiety and higher satisfaction with the simplified form, but no change in comprehension levels [15]. A systematic review of 42 trials studying alterations in the informed consent process found that multimedia approaches (slide presentations, videos, interactive computer programs) and readability enhancements had little effect [14]. Koyfman et al. demonstrated that local IRB reviews of ICFs for cooperative-group trials result in significant lengthening of the consent forms, and worsening of readability, indicating that such reviews fail in their goal to enhance human subject autonomy [25]. In our analysis, ICFs from investigator-initiated trials were nearly half the length of those from industry-sponsored trials, without any apparent improvement in comprehension. Because the readability of all the ICFs was poor, bringing their language to a more readable level might at least improve the patients’ subjective satisfaction. Shortening words and sentences, using bullet points or familiar words and phrases, and then evaluating the impact of these changes on the estimation of readability have been advocated for this purpose [26]. Using this pilot experience, our group is planning to study a cohort of patients treated on trials with the newly revised, shortened ICF templates according to the National Cancer Institute initiative. In fact, a simple single-page summary of the study that highlights its design, experimental nature, uncertain benefits and potential risks, might well supplement the full ICF.

**Fig 3:**
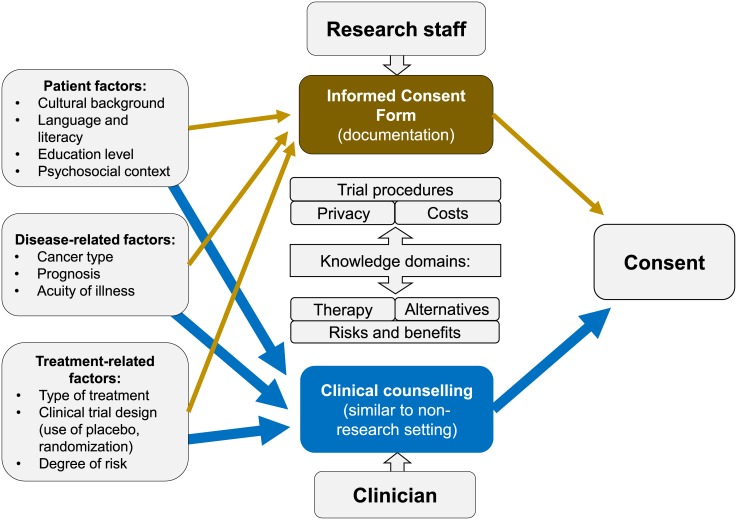
A conceptual model, emphasizing the complementary nature of the informed consent form and clinical counselling, with regard to knowledge domains necessary for complete informed consent.

The failure to improve on the efficacy of ICF’s over the past 20 years indicate that an adjustment to the classical conceptual framework of informed consent may be needed, de-emphasizing the role of ICF as a replacement for medical counselling (Fig 3). The ICF is a part of the informed consent process, designed to complement, not replace, a discussion between researchers and subjects, but serves primarily as documentation rather than an educational tool. Our results suggest that in-person discussions should focus on those aspects of consent most often poorly conveyed through the ICF—specifically the experimental nature of treatment, associated risks, benefits, and alternatives, similar to counseling regarding standard treatment options in clinical oncology. These knowledge domains may be best suited for face-to-face discussions with expert clinicians, which are easily adaptable to patient’s level of education and cultural characteristics, and can include an interpreter for patients not proficient in English. Such conversations are known to omit some components of the informed consent, and their completeness and linguistic complexity varies considerably among clinicians, so other critical knowledge domains may be delegated to an ICF review with non-clinical research staff [27]. Nishimura et al. conducted a meta-analysis of 22 various interventions, finding that extended discussion during the consent process was associated with increased comprehension scores, particularly in studies that used real clinical trial participants rather than simulations [28]. Leveraging the complementarity between the ICF and in-person discussion, as well as roles of clinicians and non-clinical research staff might provide an attractive approach to improving patient understanding.

Our study had several important limitations. Only patients participating in qualifying trials and receiving active cancer therapy were included, and while all screened patients agreed to participate, we did not evaluate potential selection bias related to early loss of follow-up, early withdrawals from treatment trials, or failure to screen all clinical trial participants in the participating institutions. Such selection bias might affect both overall distribution of variables, and the identified associations. Some patients enrolled in our study many weeks after consenting to their treatment trial, although we did not find a significant correlation between the time between the two consents and the QuIC-A score. The cohort was largely homogeneous with regard to race/ethnicity and native language, and statistical power is limited by sample size. Therefore, the observed associations between the objective understanding of ICF and education level should be viewed as hypothesis-generating rather than definitive. Because only a minority of research participants at RIHCCC enrolled in this trial, we cannot rule out selection bias related to patients’ characteristics which might also affect understanding of the ICF. In particular, the cohort contained mainly patients with advanced gastrointestinal malignancies, whose care is provided by specialized staff oncologists and for whom there were often limited therapeutic options, thus potentially exacerbating their sense that treatment on a clinical trial was their best, if not only, alternative. Furthermore, the QuIC instrument includes only closed questions, which offer limited insight into comprehension. A qualitative assessment using open questions might help to elucidate at which step the informed consent process is failing to convey the essential information about therapy in an understandable manner. Finally, we did not evaluate the contents of the ICFs for studies included in this analysis, although those had been reviewed by appropriate institutional review boards with regard to adequate explanation of benefits and risks of trial participation.

## Conclusion

In conclusion, providing true informed consent remains a major challenge for investigators and patients involved in cancer clinical trials, which persists in the era of biologic, targeted and immune therapies. The readability of ICFs, measured by the reading grade and Reading Ease Score, remains outside of the FDA guidelines [23], contributing to poor comprehension. As identified in our analysis, aspects of informed consent related to the actual experimental therapy are the most prone to misunderstanding, and need to be at the center of efforts to improve patient comprehension. Our results suggest that this is a challenge facing all research sponsors—industry, cooperative groups and local investigators alike. The problem is so pervasive, and correcting it so vital to both patients and research enterprise, that we need to investigate a diversity of approaches, from simplified ICFs and supplemental online or printed resources, to greater reliance on direct communication between oncologists and their patients.

## Supporting information

S1 Appendix Clinical trial protocol for BrUOG-274. (PDF)

S1 Dataset Dataset from the BrUOG-274 clinical trial. (PDF)
