# Supplementary material for: Informed consent in oncology clinical trials: A Brown University Oncology Research Group prospective cross-sectional pilot study
Source: PLoS One. 2017 Feb 24;12(2):e0172957. doi: 10.1371/journal.pone.0172957 (PMC5325585; doi:10.1371/journal.pone.0172957)
Supplement: S1 Appendix — (PDF) [file pone.0172957.s001.pdf]

BrUOG 274

Do Patients Participating In Oncology Clinical Trials Understand the Informed Consent Form?

A Brown University Oncology Research Group Study

<u>Principal Investigator</u>
Andrew Schumacher, MSHCE

Protocol Draft: 2/8/2012
Amendment #1 9-28-12
Amendment #2 11-8-12

1.0 OBJECTIVES:

1.1 Primary Objective

1.1.1 To evaluate if patients participating in oncology clinical trials understand the important elements of the informed consent forms.

1.2 Secondary Objective:

1.2.1 To evaluate if age, race, native language, education level, type of cancer, consent form length and source of clinical trial (cooperative group, Pharma or investigator initiated) affect patient comprehension.

2.0 BACKGROUND:

Historical perspective of informed consent: Obtaining informed consent as part of a clinical study is a cornerstone of current day medical ethics; however, this has not always been the case. The development of the informed consent has been necessitated out of abuses committed in the name of medicine. Two important documents that resulted from these abuses are the Nuremberg Code and the Belmont Report. Both of these documents identified patient autonomy as an essential element of informed consent.[1, 2] Autonomy refers to a patient making a decision to participate in a clinical study by their own free will. Knowledge of the purpose, risks, benefits and alternatives is central to an individual making an informed decision about clinical trial participation.

Tom Beauchamp attempts to identify what is an informed consent and like the Belmont Report and Nuremberg Code identifies patient autonomy as a key element.[3] He goes onto state that "[a] person gives an informed consent…if and only if the person, with substantial understanding and in substantial absence of control by others, intentionally authorizes a health professional to do something."[3 (517 -518)] Beauchamp places conditions that need to be met so patient autonomy can be achieved and the condition of understanding brings to the forefront the issue of therapeutic misconception.

The dual role of physician-investigator and therapeutic misconception: Applebaum et al coined the term "therapeutic misconception" in a study of informed consent in psychiatric research.[4] The Applebaum et al study identifies therapeutic misconception as the conflation of the goals of medical care and those of the research study by the study participants.[4, 5] The goal of medical care is to provide individualized, optimized care for patients; whereas the goal of a clinical research study is to answer the question at the heart of the study resulting in generalizable information that benefits future patients.[5] Therapeutic misconception is ethically concerning because of a number of issues like the validity of informed consent and blurring the line in the patient-care provider relationship.

It has been suggested that the confusion regarding the aims of medical care and the aims of the research occur due to a lack of understanding by the research participants. In a study conducted by Joffe et al, 25% of the research participants in cancer clinical trials surveyed did not recognize that the main purpose of the study was to benefit future patients, 70% the unproven nature, and 74% non-standard treatment.[6] This lack of

understanding by research participants is concerning as it may compromise the validity of the informed consent.[7] There is little consensus to what constitutes adequate understanding by a research participant; however he or she should have some comprehension regarding the information and its bearing on his or her situation.[7] Without sufficient comprehension about the information provided the research participant cannot meet the condition of understanding that is vital to a valid informed consent.

In the clinical research setting the line defining physicians and investigators is often blurred as the same person may be acting in both roles. In this dual role that many physician-investigators take on in clinical research, they have commitments to protect and help the patients, as well as relying on the patient's continued participation in the research to obtain the generalizable information to benefit future patients.[8]

The dual role of physician-investigator has the potential to expose the patient to the therapeutic misconception. When patients enter into a clinical research study he or she could assume that the goal of the physician-investigator is the same as that as a physician only acting in one capacity, which would be providing the optimal individualized care for the patient. The physician-investigator has an obligation to the clinical research study and future patients to obtain the generalizable knowledge.[8] Patients, whether they be research participants or not, have an interest in understanding and determining the relationships in which they enter and this distinction between physician and physician-investigator may go unnoticed by patients who agree to enter into a research and as a result they may not understand the change in the relationship with his or her care provider.[8]

The line is also blurred by the setting in which the clinical trials are conducted. Most often clinical trials are conducted in the same setting where standard medical care is provided using the same instruments and procedure rooms. The ambiguousness between research procedures and standard medical care perpetuates the therapeutic orientation of clinical research and may make it difficult for research participants to decipher a clinical trial from standard medical care.[9] Additionally; the clinical orientation of a clinical trial is reinforced by the manner in which the trials have been presented to the general public. The advertisements directed to recruit potential participants appeal to people who are suffering from illness or disease and are seeking therapy, rather than appealing to the altruistic motivation of to contribute to the generalizable information obtained by the study that may benefit future patients.[9]

<u>Basic elements of informed consent:</u> The federal regulation, issued by the Department of Health and Human Services, establishes 8 basic elements of informed consent and states: "(t)he information that is given to the subject or the representative shall be in language understandable to the subject or the representative."[10]

Federal regulations, issued by the Department of Health and Human Services establish the following 8 basic elements of the informed consent:[10]

(1) A statement that the study involves research, an explanation of the purposes of the research and the expected duration of the subject's participation, a description of the

procedures to be followed, and identification of any procedures which are experimental;

(2) A description of any reasonably foreseeable risks or discomforts to the subject;

(3) A description of any benefits to the subject or to others which may reasonably be expected from the research;

(4) A disclosure of appropriate alternative procedures or courses of treatment, if any, that might be advantageous to the subject;

(5) A statement describing the extent, if any, to which confidentiality of records identifying the subject will be maintained;

(6) For research involving more than minimal risk, an explanation as to whether any compensation and an explanation as to whether any medical treatments are available if injury occurs and, if so, what they consist of, or where further information may be obtained;

(7) An explanation of whom to contact for answers to pertinent questions about the research and research subjects' rights, and whom to contact in the event of a research-related injury to the subject; and

(8) A statement that participation is voluntary, refusal to participate will involve no penalty or loss of benefits to which the subject is otherwise entitled, and the subject may discontinue participation at any time without penalty or loss of benefits to which the subject is otherwise entitled.

Quality of Informed Consent (QuIC) survey: The QuIC survey was utilized in publications in 2001 to assess the quality of the informed consent and assess a participant's comprehension of the informed consent.[6] The QuIC survey covers these eight basic elements of the informed consent as identified by the United States federal regulations. In the complete QuIC survey, Part A is designed to measure patients' knowledge on the basics of clinical trials and Part B is comprised of 14 questions that patients rate their understanding of basics of clinical trials.[11,12] The QuIC questionnaire has been validated by Joffe et al in a cross-sectional survey of cancer clinical trials.[6,12]

Increasing complexity of informed Consent: Since Joffe et al conducted the original study in 2001; consent documents have become increasingly complex and lengthy. Studies by LoVerde et al and Berger et al showed that the informed consent document has lengthened significantly since 1982.[13-14] Some of this increased length can be attributed to a more in-depth disclosure of potential risks, study procedures, and confidentiality; however much of the language added is regulatory and legal in nature and used to protect the institution conducting the research. Not only do these regulatory/legal clauses increase length of the informed consent document, they also decrease the readability.[15] Although there is little data, the data available suggests that people are unlikely to read documents >1000 words (approximately 4 pages) in length.[14-15] In a study by Sharp, the average length of a consent document (both cooperative group and industry sponsored) was 2709 words or about 11 pages, more than twice the length that people are likely to read.[15] The increasing length of consent documents may discourage

people from reading the consent documents thoroughly and compromise the communication of required information to the patient and the validity of the informed consent.[14-15]

<u>Protocol Rationale:</u> Informed consents have become increasing lengthy and complex. It is not unusual for an Oncology Clinical Research informed consent to be greater than 20 pages. We will utilize the QuIC-A survey to evaluate patient understanding of the basic elements of informed consent required by federal regulations. The QuIC-A survey has been condensed from 20 questions to 14 questions by eliminating the 6 phase specific questions. The remaining 14 questions are designed irrespective of phase and address the basic elements of informed consent.

We hypothesize that as informed consents have become more lengthy and complicated, in part due to required regulatory language and legal clauses inserted to protect the institution performing research, patient comprehension of the basic elements of informed consent has been hindered.

3.0 ELIGIBILITY:

3.1 Patients ≥ 18 years of age

3.2 Patients are receiving active treatment on an oncology clinical trial that utilizes chemotherapy, radiation, targeted agents, biologic therapy or hormonal therapy.

3.3 Patients must have been consented in English to an oncology treatment clinical study

4.0 STUDY DESIGN:

The process for this trial will be as follows: All patients entering on this trial will have been enrolled on an oncology clinical trial and are receiving active anti-cancer treatment on that study. Patients will be asked by a member of the research team if they would like to participate in this study evaluating patients' comprehension of the informed consent for the oncology treatment study that they are participating in. Patients will be consented and will be told that, at their next clinical visit, they will be given the questionnaire to complete. (Having time between consenting to this quality improvement study and the questionnaire completion will limit patient's confusion. It will facilitate patients being able to focus on the previously signed treatment consent form.)

When patients return to clinic at their next clinical visit, they will be given the option to complete the questionnaire on site after being given the document or have the ability to mail the completed questionnaire into the research office once completed. If the questionnaire is not returned within 2-weeks, the participant will be approached again during their normal clinical visit and asked if they still wish to participate in this study. If they still agree the participant will be given a second questionnaire, which they can either complete on site or they are again given the opportunity to mail the completed questionnaire into the research office. If a questionnaire is not received within 1-month of dispersing the second questionnaire the participant will be viewed as a non-responder unless a questionnaire is received.

Information that will be obtained on all patients by the study staff when registering patients for this protocol including the following:

- Age
- Sex
- Native language (if English is a second language)
- Race
- Highest Level of Education
- Cancer Type
- Phase of clinical trial
- Type of sponsor (i.e. cooperative group, pharmaceutical company, or investigator initiated)
- Time interval in months between signing the informed consent for the oncology treatment study to the time interval to signing informed consent for this comprehension study
- Institution in which patient is participating in oncology treatment study
- Length and readability statistics of the consent form

4.1: Questionnaire

5.0 REGULATORY AND ETHICAL ISSUES

5.1 IRB Approval

Questionnaires will not be given or sent to patients until the Internal Review Board (IRB) of the participating institution(s) or applicable national IRB has reviewed and approved this study.

5.2 Informed Consent

Informed consent will be obtained in writing from all patients prior to entry into the study. A sample Informed Consent document is included as Appendix A.

5.3 Patient Confidentiality

Those knowing the identity of patients participating in the study will be strictly controlled. The research personnel will keep a log to record each patient that agrees to answer the questionnaire. The patient's initials are the only identifiers that will be listed on the data record for the study. The log and other study files will be kept in a secure location at the site.

Only anonymized or summary data will be shown or published.

The study will be conducted in accordance with the requirements outlined by The Health Insurance Portability and Accountability Act (HIPAA).

5.4 Data Safety Monitoring Board

All trials initiated by the Brown University Oncology Research Group (BrUOG) are subject to oversight by the Data Safety Monitoring Board (DSMB). This board meets at least two times per year with any additional meetings scheduled as needed. This is a non-treatment study so adverse events are not expected however, the BrUOG DSMB will still

monitor this study. Following each DSMB meeting, provide the study leadership with written information concerning findings for the trial as a whole related to any study difficulties observed and any relevant recommendations related to continuing, changing, or terminating the trial. The study leadership will provide information and relevant recommendations to the local principal investigators to be shared with their IRB(s).

6.0 Patient Registration:

All patients will be registered through the Brown University Oncology Research Group Central Office. Eligibility Checklist with supporting documentation (demographics form), On Study Form and the first and last page of the signed Patient Consent Form must be faxed emailed or scanned to the BrUOG Central Office, , at the time of registration and prior to patient treatment.

Fax: (401) 863-3820,
Email: Kayla_rosati@brown.edu

Brown University Oncology Research Group will provide case report forms, included in the appendix, for the recording and collection of data. In the event of corrections, each correction will be initialed and dated by the person making the correction. The investigator will sign the case reports to indicate that, to his/her knowledge, they are complete and accurate. Case report forms, flow sheets, off-study forms and follow-up forms should be mailed / faxed to:

Brown University Oncology Research Group
Phone: (401) 863-3000, Fax: (401) 863-3820
Email: Kayla_rosati@brown.edu

All supporting data must be sent in with the corresponding BrUOG forms.

7.0 STATISTICAL ANALYSIS:

The questionnaires will be scored with respect to the correct answers. Multivariate analysis will be performed to evaluate the effect of age, sex, native language, race, highest level of education, cancer type, phase of clinical trial, type of sponsor (cooperative group, pharmaceutical, BrUOG) or institution in which patient is participating in oncology treatment study.

8.0 REFERENCES

1) Annas, G., Grodin, M. (2008). **"The Nuremberg Code"**. In Emanuel, E., Grady, C., Crouch, R., Lie, R. Miller, F. Wendler, D. (Eds) *The Oxford Textbook of Clinical Research Ethics* (pg 136-140) New York: Oxford University Press.

2) Jones, J. (2008). **"The Tuskegee Syphilis Experiment."** In Emanuel, E., Grady, C., Crouch, R., Lie, R. Miller, F. Wendler, D. (Eds) *The Oxford Textbook of Clinical Research Ethics* (pg 86-96) New York: Oxford University Press.

3) Beauchamp T. **"Informed Consent: Its History, Meaning, and Present Challenges."** *Cambridge Quarterly of Healthcare Ethics*. 2011; **20**: 515-523.

4) Applebaum P, Roth LH, Lidz C. **"The Therapeutic Misconception: Informed Consent in Psychatric Research."** *Int J Law Psychiatry*. 1982; **5**: 319-329.

5) de Melo-Martin I, Ho A. **"Beyond Informed Consent: The Therapeutic Misconception."** *J Med Ethics*. 2008; **34**: 202-205

6) Joffe S, Cook EF, Cleary PD, Clark JW, Weeks JC. **"Quality of Informed Consent in Cancer Clinical Trials: A Cross-Sectional Survey."** *Lancet*. 2001; **358**: 1772-1777.

7) Del Carmen M, Joffe S. **"Informed Consent for Medical Treatment and Research: A Review."** *The Oncologist*. 2005; **10**: 636-641.

8) Wendler D, Grady C. **"What Should Research Participants Understand to Understand They Are Participants in Research?"** *Bioethics*. 2008; **22**; 4: 203-208.

9) Miller FG, Rosenstein DL. "**The Therapeutic Orientation to Clinical Trials**." *N Engl J Med*. **2003**; 348: 1383-1386

10) Protection of Human Subjects, 45 CFR §46. Available at http://ohsr.od.nih.gov/guidelines/45cfr46.html#46.116 Accessed December 3, 2011.

11) Bergenmar M, Molin C, Wilking N, Brandberg Y. **"Knowledge and Understanding Among Cancer Patients Consenting to Participate in Clinical Trials."** *European Journal of Cancer*. **2008**; 44: 2627-2633

12) Joffe S., Cook EF, Cleary PD, Clark JW, Weeks JC. **"Quality of Informed Consent: a New Measure of Understanding Among Research Subjects."** *J Natl Cancer Inst*. **2001**; 93: 139-147

13) LoVerde ME, Prochazka AV, Byyny RL. **“Research consent forms:continued unreadability and increasing length.”** *J Gen Intern Med* **1989**; 4:410–412.

14) Berger o, Grønberg BH, Sand K, Kaasa S, Loge JH. **“The length of consent documents in oncological trials is doubled in twenty years.”** *Annals of Oncology.* **2009**; 20: 379–385.

15) Sharp, SM. **“Consent Documents for Oncology Trials: Does Anybody Read These Things?”***Am J Clin Oncol*. **2004**; 27: 570-575.

Appendix A

**Affiliate**	☒ Rhode Island Hospital	☒ The Miriam Hospital
	☐ Bradley Hospital	☐ Newport Hospital

Agreement to Participate in a Research Study
And Authorization for Use and Disclosure of Information

______________ Committee #

_________________________ Name of Study Volunteer

Do Patients Participating In Oncology Clinical Trials Understand the Informed Consent Form?

You are being asked to take part in a research study. All research studies carried out at _____________ institutions are covered by rules of the Federal government as well as rules of the State and_____________. Under these rules, the researcher will first explain the study, and then he or she will ask you to participate. You will be asked to sign this agreement which states that the study has been explained, that your questions have been answered, and that you agree to participate.

The researcher will explain the purpose of the study. He or she will explain how the study will be carried out and what you will be expected to do. The researcher will also explain the possible risks and possible benefits of being in the study. You should ask the researcher any questions you have about any of these things before you decide whether you wish to take part in the study. This process is called informed consent.

This form also explains the research study. Please read the form and talk to the researcher about any questions you may have. Then, if you decide to be in the study, please sign and date this form in front of the person who explained the study to you. You will be given a copy of this form to keep.

1. <u>Purpose and Explanation of the Study</u>

You are being asked to participate in this survey because you are enrolled in a cancer clinical trial. The purpose of this study is to assess your understanding of clinical trials and to help researchers understand which areas of the informed consent need better explanation for future cancer clinical trial patients.

The study involves a one-time survey, which will have questions about clinical trials and general demographic questions. The survey takes about 15 minutes to complete. You may leave any questions blank that you do not want to answer. You decide whether or not you want to complete the survey. Participation is voluntary. If you decide not to participate, it will not affect the health care services that you normally receive.

If you agree to complete the survey, please do NOT write your name on it. After you finish filling it out, please put the survey in the return envelope provided.

There will be no financial cost to you or your health plan/insurance company for taking part in this study.

If you have any questions please contact Andrew Schumacher, the Principal Investigator, at (401) 444 -3234 or the Research Office at (401XXX-XXXX.

2. <u>Risks and Benefits</u>

The greatest risk to you is the release of information from your health records. We will do our best to make sure that your personal information will be kept private.

There are no direct benefits to you for taking part in this research. The information gained from this study may benefit cancer patients in the future.

3. <u>Rights and Complaints</u>

If you have any complaints about your taking part in this study, or would like more facts about the rules for research studies, or the rights of people who take part in research studies, you may contact _______________, in the ________ Office of Research Administration, at (401) XXX-XXXX.

4. <u>Confidentiality</u>

The section at the end of this document called "Research Authorization for Use and Disclosure of Information" provides detailed information about how the information learned about you during this study will be used and shared. More generally, all of your records from this study will be treated as private health care records. The records will be protected according to the rules of __________. The ____________ privacy practices and policies are based on the rules about protection of private health care information contained in Rhode Island law and in the Federal Health Insurance Portability and Accountability Act of 1996 and its regulations ("HIPAA"). The privacy practices of __________ and of the people who provide services at or with ___________ are explained in more detail in the _________ Joint Privacy Notice (the "Privacy Notice'), which will be given to you.

You should also know that there are times when the law might require or permit _______ to release your health information without your permission. The Privacy Notice explains when this might happen. To give you some examples, State law requires health care workers to report abuse or neglect of children to the Department of Children, Youth and Families (DCYF). State law also requires health care workers to report abuse or neglect of people age 60 and older to the Department of Elderly Affairs.

5. <u>Research authorization for use and disclosure of information</u>.

The purpose of this section of the document is to provide you with some more information about how the information learned about you during the study will be used and shared.

We understand that your medical information is very personal and we will work hard to keep it private. If you sign this form you consent to participate in this research study and are giving us permission to use and share your personal health information in the ways described in this form.

Understandings and notifications
The main purpose of permitting the use and release of your information is to allow the research project to be conducted and to ensure that the information relating to that research is available to all parties who may need it for research purposes. Your information may also be used as necessary for your research-related treatment, to collect payment for your research-related treatment (when applicable), and to run the business operations of the hospital.

All health care providers are required to protect the privacy of your information. However, most persons or entities (i.e., businesses, organizations) that are not health care providers are not bound by law to protect the privacy of your information. You understand that if the person or entity that receives your information is not a health care provider bound to protect your privacy, such person or entity might re-release your health information.

You have the right to refuse to sign this form. If you do not sign this form, none of your health care outside the study, or the payment for your health care, or your health care benefits will be affected. However, if you do not sign this form, you will not be able to enroll in the research study described in this form, and you will not receive treatment as a study participant.

If you sign this consent form, you may withdraw from the study at any time. However, if you do not want the researchers to use or disclose any further information in this study you must cancel permission in writing and may do so at any time. If you cancel your permission, you will stop taking part in the study and no new information will be collected about you. However, if you cancel your permission, it will not apply to actions already taken or information already collected about you by the hospital or the researchers before you canceled your permission. This information or action may be needed to complete analysis and reports of this research. This permission will never expire unless you cancel it. To cancel this permission, please write to Andrew Schumacher, the Principal Investigator, at Rhode Island Hospital, APC Building, Room 131, Providence, RI 02903.

If after you have signed this form you have any questions relating to your rights, please contact ______________ in the Office of Research Administration, (401) XXX-XXXX.

Uses and releases covered by this authorization (permission)
Who will release, receive, and/or use your information? This form will allow the following person(s), class(es) of persons, and/or organization(s)* to release, use, and receive the information listed below in connection with this Study, or as required by law:

- ☒ Every research site for this study, including this hospital, and including each site's research staff and medical staff
- ☒ The following research sponsors and the people and companies that they use to oversee, administer, or conduct the research: Brown University Oncology Research Group
- ☒ The United States Food and Drug Administration, the Department of Health and Human Services, the Office of Inspector General, and the Office of Civil Rights.

☒ The members and staff of the Institutional Review Board(s) or Ethics Committee(s) that approves this study
☒ Principal Investigator and other Investigators
☒ Study Coordinator
☒ Additional members of the Research Team
☐ The Patient Advocate or Research Volunteer Protector: ____________________
☒ Members of the hospital's administrative staff responsible for administering clinical trials and other research activities
☐ Contract Research Organization (A contract research organization is an independent organization that agrees to oversee and make possible, various aspects of the clinical research process for the research sponsor.)
☒ Data and Safety Monitoring Boards and others that monitor the conduct of the Study, for example a Clinical Events Committee
☒ The members and staff of the hospitals affiliated Privacy Board (if such a board is used)
☐ Others (as described below)____

* If, during the course of the research, one of the companies or institutions listed above merges with or is purchased by another company or institution, this permission to use or release protected health information in the research will extend to the new company or institution.

☒ The entire research record and any medical records held by the hospital may be used and released.

SIGNATURE

I have read this informed consent and authorization form. ALL OF MY QUESTIONS HAVE BEEN SATISFACTORILY ANSWERED, AND I WANT TO TAKE PART IN THIS RESEARCH STUDY.

By signing below, I give my permission to participate in this research study and for the described uses and releases of information. *I also confirm that I have been now or previously given a copy of the _____________ Privacy notice*

This informed consent document expires on _____________.
DO NOT sign this document after this expiration date

____ ________ ____________

Signature of study volunteer/authorized representative* Date and Time when signed

I was present during the consent PROCESS AND signing of this agreement above by the study volunteer or authorized representative

____ ______________

Signature of witness (required if consent is presented orally or at the request of the IRB) Date

I ASSURE THAT I HAVE FULLY EXPLAINED TO THE ABOVE STUDY VOLUNTEER/AUTHORIZED REPRESENTATIVE, THE NATURE AND PURPOSE, PROCEDURES AND THE POSSIBLE RISK AND POTENTIAL BENEFITS OF THIS RESEARCH STUDY.

____ ________ __________

Signature of researcher or designate Date and Time when signed

* If signed by agent other than study volunteer, please explain below.

____

____

Documentation that a copy of this Informed Consent was given to the research participant is a Federal requirement. Prior to making a copy of the signed and dated Informed Consent please check appropriate box(es) as applicable to indicate copy provided to:

☐ Study Volunteer ☐ Medical Record ☐ Researcher ☐ Other (Specify)

Appendix B

Dear XXXX,

You are receiving this letter because you have agreed to participate in a survey study called "**Do Patients Participating In Oncology Clinical Trials Understand the Informed Consent Form?**"

The attached survey involves answering some general questions about clinical trials. The survey takes about 15 minutes to complete. The purpose of this study is to assess your understanding of clinical trials and to help researchers understand which areas of the informed consent need better explanation for future cancer clinical trial patients. Your participation is completely voluntary. You do not have to answer any question you do not want to answer. After you finish filling it out, please put the survey in the return envelope provided.

If you do not wish to participate, you may contact the Oncology Clinical Research office at (401)XXX-XXXX or return the blank survey, in the provided return envelope, noting that you do not wish to participate.

If you have any questions please contact Andrew Schumacher, the Principal Investigator, at (401) 444 -3234.

Please keep this letter for your records. Thank you for your participation.

Regards,

Quality of Informed Consent

INSTRUCTIONS: Below you will find several statements about cancer clinical trials (otherwise known as cancer research studies). Thinking about your clinical trial, please read each statement carefully. Then tell us whether you agree with the statement, you disagree with the statement, or you are unsure about the statement by circling the appropriate response. Please respond to each statement as best you can. We are interested in your opinions.

A1.	When I signed the consent form for my current cancer therapy, I knew that I was agreeing to participate in a clinical trial	Disagree	Unsure	Agree
A2.	The main reason cancer clinical trials are done is to improve the treatment of future cancer patients.	Disagree	Unsure	Agree
A3.	I have been informed how long my participation in this clinical trial is likely to last.	Disagree	Unsure	Agree
A4.	All the treatments and procedures in my clinical trial are standard for my type of cancer.	Disagree	Unsure	Agree
A5.	The treatment being researched in my clinical trial has been proven to be the best treatment for my type of cancer.	Disagree	Unsure	Agree
A6.	Compared with standard treatments for my type of cancer, my clinical trial does not carry any additional risks or discomforts.	Disagree	Unsure	Agree
A7.	There may not be direct medical benefit to me from my participation in this clinical trial.	Disagree	Unsure	Agree
A8.	By participating in this clinical trial, I am helping the researchers learn information that may benefit future cancer patients.	Disagree	Unsure	Agree
A9.	Because I am participating in a clinical trial, it is possible that the study sponsor, various government agencies, or others who are not directly involved in may care could review my medical records.	Disagree	Unsure	Agree
A10.	My doctors did not offer me any alternatives besides treatment in this clinical trial.	Disagree	Unsure	Agree
A11.	The consent form I signed describes who will pay for treatment if I am injured or become ill as a result of participation in this clinical trial.	Disagree	Unsure	Agree
A12.	The consent form I signed lists the name of the person (or persons) whom I should contact if I have any questions or concerns about the clinical trial.	Disagree	Unsure	Agree
A13.	If I had not wanted to participate in this clinical trial, I could have declined to sign the consent form.	Disagree	Unsure	Agree
A14.	I will have to remain in the clinical trial even if I decide someday that I want to withdraw.	Disagree	Unsure	Agree

BrUOG 274 Do Patients Participating In Oncology Clinical Trials Understand the Informed Consent Form?
Demographics Form: To be completed by research staff

Patient's Initials: ______________________________ **Age**: ______________________

☐ Male ☐ Female

Native Language:
☐ Cambodian
☐ Cape Verdean
☐ English
☐ French
☐ French Creole
☐ Hmong
☐ Laotian
☐ Portuguese
☐ Russian
☐ Simplified Chinese
☐ Spanish
☐ Traditional Chinese
☐ Vietnamese
☐ Other

Educational Achievement:
☐ High School Diploma
☐ Associates Degree
☐ Bachelors Degree
☐ Masters Degree
☐ Doctorate (PhD, MD, JD etc.)
☐ Other ________________________

Race:
☐ White (non-Hispanic)
☐ Hispanic/Latino
☐ African American
☐ Asian
☐ Native American
☐ Pacific Islander
☐ Other ________________________

Diagnosis: ________________________ **Phase of Trial**: ☐ **I** ☐ **II** ☐ **III** ☐ **IV**

Name of Institution: ____

Date of Clinical Trial Consent: ____________________________________

Type of Trial:
☐ Cooperative Group
☐ Industry Sponsored
☐ Physician Initiated (BrUOG)

Clinical Trial Consent Form:
Number of pages ______________________________
Flesch- Kincaid Grade Level ____________________
Flesch Reading Ease ___________________________

BrUOG 274 Do Patients Participating In Oncology Clinical Trials Understand the Informed Consent Form?
Eligibility Checklist

Please document to confirm eligibility

______ Patients $\geq$ 18 years of age

______ Patients are receiving active treatment on an oncology clinical trial that utilizes chemotherapy, radiation, targeted agents, biologic therapy or hormonal therapy.

______Patients must have been consented in English to an oncology treatment clinical study

_________ Signed informed consent (this study)   Date signed:___/______/______

IRB approval date of protocol: ______/____/_______

Hospital where patient will be treated: ____________

Date patient will get teaching on: ____________

Primary Physician: _____________

The patient (Initials only) ___________________has met the above protocol criteria on

_____________________ and is assigned BrUOG sequence number ____________

Your signature: ____

PLEASE FAX, SCAN OR EMAIL THIS FORM ALONG WITH: DEMOGRAPHICS FORM, THE FIRST/LAST PAGE OF THE ICF, AND AN ONCOLOGY TREATMENT NOTE SHOWING PT IS RECEIVING ACTIVE TREATMENT,

Brown University Oncology Research Group
Phone: (401) 863-3000, Fax: (401) 863-3820
Email: Kayla_rosati@brown.edu
