# Supplementary material for: Informed consent in oncology clinical trials: A Brown University Oncology Research Group prospective cross-sectional pilot study
Source: PLoS One. 2017 Feb 24;12(2):e0172957. doi: 10.1371/journal.pone.0172957 (PMC5325585; doi:10.1371/journal.pone.0172957)
Supplement: S1 Dataset — (PDF) [file pone.0172957.s002.pdf]

ID	Age	Male sex	Non-English language	Race	Education	Tumor	Days between consents	Trial phase	Trial sponsor	Treatment	ICF pages	ICF reading grade	ICF Flesch-Kincaid	q1	q2	q3	q4	q5	q6	q7	q8	q9	q10	q11	q12	q13	q14
1	58	0	0	0	Bachelors	Pancreatic	33	3	Industry	Biolo	25	10.8	47.1	0	0	0	0	0	0	0	0	0	1	0	0	0	1
2	73	0	0	0	HSD	Pancreatic	59	3	Industry	Biolo	25	10.8	47.1	0	0	1	9	9	9	0	0	0	0	0	0	0	1
3	63	1	0	0	Bachelors	Pancreatic	56	1	COG	Biolo	19	10.5	52.6	0	0	0	0	0	9	1	0	0	0	0	0	0	1
4	69	0	0	0	HSD	Pancreatic	38	1	COG	Biolo	19	10.5	52.6	0	9	9	0	0	1	0	0	0	1	9	0	0	1
5	65	0	0	0	HSD	Colorectal	73	1	IIS	Chemo	12	11.0	51.2	0	9	0	9	0	9	0	0	0	1	0	9	0	1
6	65	0	0	0	Masters	Pancreatic	56	3	Industry	Biolo	26	10.8	47.1	0	0	1	1	1	1	0	0	0	1	0	0	0	1
7	54	1	0	0	Masters	Pancreatic	21	1	COG	Biolo	19	10.5	52.6	0	9	9	0	0	9	9	0	0	1	9	0	0	1
8	61	0	0	0	Associate	Lung	271	3	Industry	Immuno	17	12.2	47.2	0	0	0	0	9	0	0	0	0	1	9	0	0	1
9	43	1	0	0	Bachelors	Melanoma	42	3	COG	Immuno	28	10.9	50.9														
10	43	0	0	0	HSD	Colorectal	50	2	IIS	Chemo	17	12.2	48.0	0	0	0	0	0	0	0	0	0	1	9	0	0	1
11	64	1	0	0	HSD	Pancreatic	177	3	Industry	Biolo	26	10.8	47.1	0	0	0	0	9	9	0	0	0	1	0	0	0	1
12	63	1	0	0	Masters	Renal	31	1	Industry	Biolo	21	11.7	49.2	0	0	9	9	9	1	1	0	9	1	0	0	0	1
13	82	1	0	0	HSD	Colorectal	40	2	Industry	Biolo	22	11.4	50.3	0	0	0	0	0	0	0	0	0	9	0	0	0	1
14	72	1	0	0	Less than HS	Esophageal	138	2	IIS	Chemo	9	11.4	47.9	0	0	0	0	0	9	0	0	0	0	0	0	0	0
15	65	1	0	0	Masters	Colorectal	29	2	IIS	Chemo	17	12.2	48.0	0	0	0	0	9	0	1	0	0	0	0	0	0	1
16	40	0	0	0	Bachelors	Breast	118	3	COG	Chemo	22	9.9	57.5	0	0	0	0	0	0	0	0	0	1	0	0	0	1
17	55	0	0	0	HSD	Lung	78	3	COG	Target	18	10.0	54.3	0	0	0	0	1	0	0	0	0	1	0	0	0	1
18	55	1	0	0	Associate	Colorectal	28	2	IIS	Chemo	12	11.1	50.2	0	0	0	9	9	0	0	0	0	1	0	0	0	1
19	67	0	0	0	Bachelors	Pancreatic	3	1	IIS	Chemo	10	10.1	52.9	0	0	0	9	0	0	0	0	0	1	0	0	0	1
20	65	1	0	0	Masters	Renal	40	1	Industry	Target	20	12.2	44.3	0	0	0	0	0	1	0	0	0	1	0	0	0	1
21	56	0	0	0	Bachelors	Colorectal	60	1	Industry	Biolo	22	11.4	50.0	0	0	0	9	9	0	0	0	0	1	0	0	0	1
22	58	1	0	0	Associate	Lung	34	1	IIS	Target	12	11.3	48.2	0	0	9	0	9	0	0	0	0	1	0	0	0	1
23	35	0	0	0	Associate	Pancreatic	46	1	IIS	Chemo	10	10.1	52.9	0	0	0	0	9	9	9	0	1	1	0	0	0	1
24	47	0	0	0	Bachelors	Pancreatic	27	1	IIS	Chemo	10	10.1	52.9	0	9	9	9	0	9	9	0	0	1	9	0	0	1
25	45	1	0	0	HSD	Colorectal	46	2	IIS	Chemo	20	12.2	48.0	0	0	0	1	1	9	0	0	0	0	0	0	0	1
26	45	1	0	2	Less than HS	Head/neck	57	1	IIS	Chemo	12	11.1	50.2	0	0	9	9	0	0	9	0	9	0	9	0	9	0
27	51	1	0	0	HSD	Colorectal	63	2	IIS	Chemo	20	12.2	48.0	0	0	0	9	9	9	0	0	0	0	0	0	0	1
28	69	0	0	0	HSD	Pancreatic	67	2	IIS	Chemo	10	10.1	52.9	0	0	9	0	0	0	0	0	0	0	0	0	0	1
29	73	1	0	2	HSD	Colorectal	70	1	IIS	Chemo	12	11.1	50.2	0	0	0	0	0	0	0	0	0	0	0	0	0	9
30	75	1	0	0	HSD	Prostate	119	2	IIS	Chemo	8	11.4	48.9	0	0	0	0	1	0	0	0	9	1	0	0	0	1
31	63	1	0	0	Associate	Pancreatic	0	1	IIS	Chemo	11	10.1	52.9	0	0	0	1	0	0	0	0	0	1	0	0	0	1
32	68	0	0	0	HSD	Colorectal	0	1	IIS	Chemo	10	10.7	52.6														
33	63	1	0	0	HSD	Cholangio	0	1	Industry	Target	16	10.4	54.4														
34	85	1	0	0	Less than HS	Prostate	69	2	Industry	Biolo	21	12.2	45.8	0	0	0	0	0	0	0	0	0	0	0	0	0	0
35	61	1	0	0	Masters	Brain	136	3	Industry	Immuno	15	11.8	46.4	0	0		0	9	1	0	0	0	1	0	0	0	1
36	46	1	0	0	Bachelors	Brain	149	3	Industry	Immuno	15	11.8	46.4	0	0	0	0	0	9	9	0	0	0	0	0	0	1
37	66	0	0	0	HSD	Breast	120	3	COG	Chemo	20	12.5	49.2	0	0	0	0	9	0	0	0	0	0	0	0	0	1
38	73	1	0	0	Bachelors	Brain	43	2	IIS	Biolo	11	10.8	51.9	0	0	0	0	1	0	0	0	0	1	1	0	0	1
39	68	1	0	0	Less than HS	Lung	20	1	IIS	Chemo	9	10.7	51.2	0	0	9	9	0	0	1	0	9	1	9	9	0	1
40	48	0	0	0	Bachelors	Pancreatic	71	1	IIS	Chemo	11	10.1	52.9	0	0	0	0	0	1	0	0	0	0	0	0	0	1
41	54	0	0	0	Bachelors	Colorectal	21	2	IIS	Biolo	9	11.6	49.8	0	0	0	0	9	1	0	9	1	1	0	0	0	1
42	51	1	0	0	HSD	Esophageal	0	3	COG	Chemo	22	10.7	55.7	0	0	0	0	0	0	0	0	0	1	9	0	0	1
43	37	0	0	0	Associate	Colorectal	0	2	IIS	Immuno	15	10.3	54.2	0	9	0	9	9	1	9	0	0	1	0	0	0	1
44	61	1	0	0	HSD	Pancreatic	119	3	COG	Target	21	11.6	50.3	0	0	0	0	9	0	0	0	0	1	0	0	0	1
45	53	0	1	1	HSD	Breast	0	1	Industry	Immuno	20	12.5	46.8	0	0	0	0	9	0	0	0	0	0	9	0	0	1
46	69	1	0	0	Bachelors	Pancreatic	68	2	IIS	Chemo	11	10.1	53.0	0	0	0	0	0	0	0	0	0	1	9	0	0	1
47	56	1	0	0	Bachelors	Pancreatic	24	2	IIS	Chemo	11	10.1	53.0	0	0	0	0	0	0	0	0	9	1	9	0	0	9
48	44	0	0	0	Associate	Pancreatic	44	2	IIS	Chemo	9	9.9	54.0	0	0	0	0	0	0	0	0	0	1	9	0	0	1
49	54	0	1	0	HSD	Breast	27	1	Industry	Immuno	20	12.4	45.9														
50	60	0	0	0	Bachelors	Pancreatic	39	2	IIS	Chemo	9	9.9	54.0	0	0	0	1	1	1	1	0	0	1	9	0	0	1
51	48	1	0	0	HSD	Brain	108	3	Industry	Immuno	26	11.9	46.6	0	0	9	9	0	9	0	0	0	0	9	9	0	1
52	67	0	0	0	HSD	Pancreatic	39	2	IIS	Chemo	9	9.9	54.0	0	0	9	0	0	9	9	0	0	9	9	0	0	1
53	69	1	0	0	Less than HS	Pancreatic	53	2	IIS	Chemo	9	9.9	54.0	0	0	0	0	0	0	0	0	0	1	0	0	0	1
54	45	1	0	0	HSD	Gastric	102	1	Industry	Immuno	21	12.5	45.6	0	0	0	1	1	1	0	0	0	1	0	0	0	1
